# Novel ALK inhibitor AZD3463 inhibits neuroblastoma growth by overcoming crizotinib resistance and inducing apoptosis

**DOI:** 10.1038/srep19423

**Published:** 2016-01-20

**Authors:** Yongfeng Wang, Long Wang, Shan Guan, Wenming Cao, Hao Wang, Zhenghu Chen, Yanling Zhao, Yang Yu, Huiyuan Zhang, Jonathan C. Pang, Sophia L. Huang, Yo Akiyama, Yifan Yang, Wenjing Sun, Xin Xu, Yan Shi, Hong Zhang, Eugene S. Kim, Jodi A. Muscal, Fengmin Lu, Jianhua Yang

**Affiliations:** 1 Department of Microbiology, Peking University Health Science Center, Beijing 100191, China; 2 Texas Children’s Cancer Center, Department of Pediatrics, Dan L. Duncan Cancer Center, Baylor College of Medicine, Houston, Texas 77030, USA; 3 Department of Pathology, University of Texas MD Anderson Cancer Center, Houston, Texas 77030, USA; 4 Department of Acupuncture, First Affiliated Hospital, Heilongjiang University of Chinese Medicine, Harbin, Heilongjiang 150040, China; 5 Department of Medical Oncology, Zhejiang Cancer Hospital, Hangzhou, Zhejiang 310022, China; 6 Department of Hepatopancreatobiliary Surgery, the Second Affiliated Hospital of Harbin Medical University, Harbin, Heilongjiang 150086, China; 7 Division of Pediatric Surgery, Michael E. DeBakey Department of Pediatric Surgery, Dan L. Duncan Cancer Center, Baylor College of Medicine, Houston, Texas 77030, USA

## Abstract

ALK receptor tyrosine kinase has been shown to be a therapeutic target in neuroblastoma. Germline ALK activating mutations are responsible for the majority of hereditary neuroblastoma and somatic ALK activating mutations are also frequently observed in sporadic cases of advanced NB. Crizotinib, a first-line therapy in the treatment of advanced non-small cell lung cancer (NSCLC) harboring ALK rearrangements, demonstrates striking efficacy against ALK-rearranged NB. However, crizotinib fails to effectively inhibit the activity of ALK when activating mutations are present within its kinase domain, as with the F1174L mutation. Here we show that a new ALK inhibitor AZD3463 effectively suppressed the proliferation of NB cell lines with wild type ALK (WT) as well as ALK activating mutations (F1174L and D1091N) by blocking the ALK-mediated PI3K/AKT/mTOR pathway and ultimately induced apoptosis and autophagy. In addition, AZD3463 enhanced the cytotoxic effects of doxorubicin on NB cells. AZD3463 also exhibited significant therapeutic efficacy on the growth of the NB tumors with WT and F1174L activating mutation ALK in orthotopic xenograft mouse models. These results indicate that AZD3463 is a promising therapeutic agent in the treatment of NB.

Originating in the sympathetic nervous system, neuroblastoma (NB) is the most frequently diagnosed embryonal malignancy of childhood^1^,^2^. It accounts for approximately 15% of all pediatric cancer-related deaths^3^,^4^,^5^, a result of metastatic progression due to poor responses to current treatment modalities. MYCN amplification and anaplastic lymphoma kinase (ALK) activation have been identified as two major oncogenic events in NB pathogenesis^6^, especially in the high-risk group. Despite improvements in treatment over recent decades, cure rates for patients with high-risk NB lag significantly behind those of other common childhood cancers^7^. Therefore, the MYCN and ALK signaling pathways are important therapeutic targets for drug development against NB.

One promising avenue for targeted therapy in NB focuses on ALK, whose expression is largely restricted to neurons and upregulated in NB. Mutations or gene amplification of ALK are associated with a NB predisposition in 15% of patients^8^. Activated ALK has been shown to promote cell growth, survival, and knockdown in NB cells inhibited proliferation^9^,^10^,^11^,^12^. In a significant proportion of cases, high ALK expression correlates with adverse outcomes in NB^11^,^13^,^14^. Increasing evidence shows that the activation of germline mutations in the ALK gene is considered to be a leading cause for most cases of hereditary NB^15^. Somatically acquired mutations are also found to be oncogenic drivers in approximately 10% of NB^9^,^10^,^11^,^15^,^16^,^17^. Out of the various somatic activating mutations identified in NB, R1275, F1174 and F1245 account for more than 85% of ALK mutations found in NB^18^,^19^. The F1174L mutation, within the kinase domain, appears to be more common than the others, leading to a higher degree of auto-phosphorylation and transforming capacity^20^,^21^. Moreover, the F1174L mutation of ALK is always associated with amplification of the MYCN^6^, predicting poor outcome in NB. Thus, NB tumors with ALK aberrations exhibit oncogenic addiction to its activity and inhibition of aberrant ALK activity is a therapeutic option for NB.

Crizotinib is an oral MET/ALK inhibitor used as first-line therapy in the treatment of advanced non-small cell lung cancer (NSCLC) harboring ALK rearrangements and has shown striking efficiency against ALK-rearranged NB, both in mouse models and in patients^3^,^22^,^23^. Early reports of crizotinib have demonstrated complete and sustained regression of xenografts harboring the R1275Q mutation but does not inhibit the growth of F1174L-positive tumors^24^. In fact, the F1174L mutation has been considered as an escape mechanism in resistant NB patients with an ALK mutation treated with crizotinib^25^. A phase 1 trial of crizotinib in patients with NB with known or unknown ALK mutations was evaluated for clinical response after treatment. Out of the 11 patients with known ALK mutations, one patient had a complete response, two had stable disease, and eight showed progressive disease. Among the 23 patients with unknown status of ALK genetic alterations, one showed complete response, five remained with stable disease, and 17 developed progressive disease^26^. Recently, two papers provided data showing that ALK tumors harboring the F1174L mutation also exhibit resistance to ceritinib, a second generation ALK inhibitor^27^,^28^. Inhibition of mutated ALK is more complicated when compared with the translocated ALK and still remains a therapeutic challenge.

AZD3463, a preclinical ALK/IGF1R inhibitor designed by AstraZeneca to overcome the acquired resistance to crizotinib, has excellent physiochemical properties. In this study, we evaluated the inhibitory effect of AZD3463 on NB cell proliferation *in vitro* and tumor growth *in vivo*. We found that AZD3463 inhibited NB cell proliferation and effectively blocked the activation of ALK-mediated PI3K/AKT/mTOR signaling in all NB cell lines tested. AZD3463 suppressed cell viability by inducing both cell apoptosis and autophagy *in vitro*. AZD3463 also significantly increased the sensitivity of NB cells to doxorubicin (Dox) treatment. Moreover, AZD3463 strongly suppressed the growth of NGP and SH-SY5Y NB tumors in orthotopic xenograft mouse models which represent both WT and F1174L oncogenic mutant ALK. Overall, the data presented here indicate that AZD3463 exhibits a potent inhibitory effect towards NB growth both *in vitro* and *in vivo*. Therefore, the results support further exploration of AZD3463 as a novel therapeutic agent in the treatment of NB.

## Results

### AZD3463 suppresses the viability and proliferation of both wild type and mutant ALK NB cells

**Figure 1:**
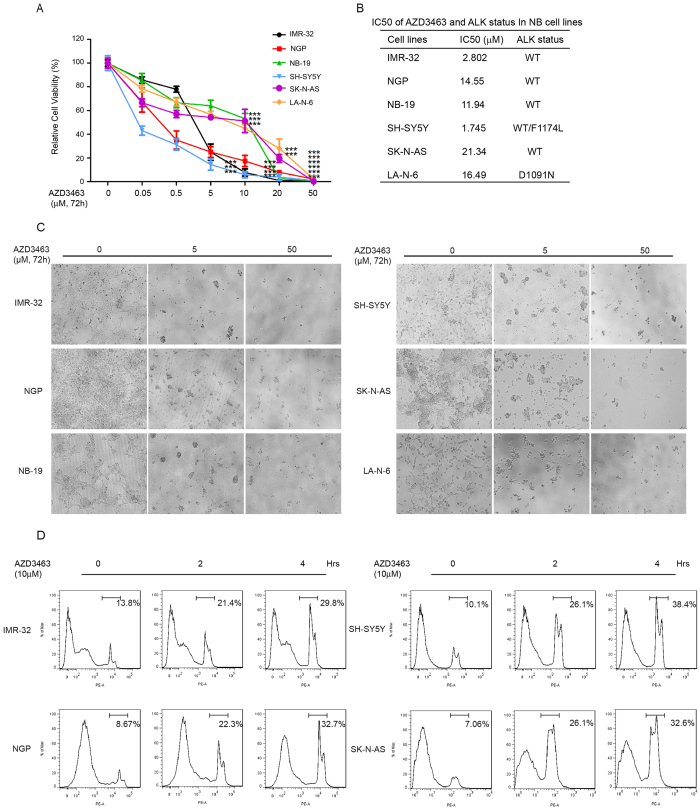
AZD3463 shows cytotoxic effects on NB cell lines. (**A**) Six NB cell lines including the established chemoresistant NB cell line LA-N-6, were treated with the increasing concentrations of AZD3463 for 72 hrs. Cell viability was then assessed by adding a mixture of 10 μL of CCK-8 and 190 μL of RPMI and measuring absorbance at 450 nm. Data is represented as % vehicle ± S.D. with *P* < 0.05 (*), *P* < 0.01 (**), or *P* < 0.001 (***) (Student’s t-test, two-tailed) as indicated. (**B**) The IC50 values of AZD3463 in each cell line listed were calculated by using Prism 5, based on the data collected in the cell viability assay. ALK status in NB cell lines was also shown. (**C**) Morphological changes of six different NB cell lines treated with increasing concentrations of AZD3463 for 72 hrs were shown. (**D**) PI staining of IMR-32, SH-SY5Y, NGP and SK-N-AS cells with the indicated concentrations of AZD3463 for 0, 2 and 4 hrs were shown. The cells were then analyzed by flow cytometry for the percentage of dead cells by incubating with PI for 15 min after AZD3463 treatment.

First of all, to explore the anti-tumor effect of AZD3463 on NB cell lines, we exposed four ALK wild type cell lines (SK-N-AS, IMR-32, NGP, NB-19) and two ALK mutant cell lines (LA-N-6 (D1091N) and SH-SY5Y (WT/F1174L))^10^,^29^ to increased concentrations of AZD3463. We then examined the cytotoxic effect of AZD3463 utilizing a CCK-8 cell proliferation assay. Notably, all cell lines tested, including ALK WT and ALK mutant cell lines, exhibited obvious sensitivity to this inhibitor. Cell morphology imaging after treatment also confirmed the cytotoxic effect of AZD3463 on these NB cell lines. We found that AZD3463 treatment significantly reduced NB cell viability in a dose-dependent manner (Fig. 1A,C), and then we calculated the IC50 of AZD3463 in different NB cell lines tested (Fig. 1B).

Consistent with the above results, the AZD3463-induced cell death was also detected in NB cell lines by flow cytometry. Treatment with AZD3463 could effectively induce cell death within 2–4 hours when compared with the DMSO control (Fig. 1D). Together, the data demonstrates that the ALK inhibitor AZD3463 can significantly suppress the viability of ALK WT and mutant NB cell lines and lead to tumor cell death in a dose-and time-dependent manners.

### AZD3463 suppresses colony formation potential of NB cells in soft agar

**Figure 2:**
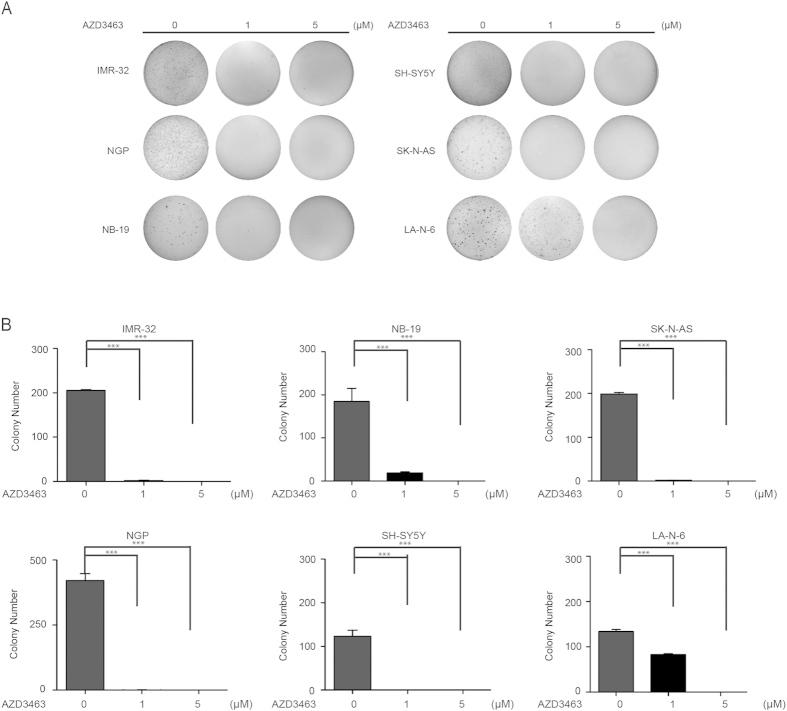
AZD3463 suppresses anchorage-independent growth of NB cells. (**A**) A panel of six NB cell lines were seeded in six-well plates with indicated concentrations of AZD3463 and agar, and grown for 2 to 3 weeks. Cells were stained with crystal violet for 4 hrs, and images were obtained. (**B**) Colonies were counted and colony numbers were represented as % vehicle ± S.D. with *P* < 0.05 (*), *P* < 0.01 (**) or *P* < 0.001 (***) (Student’s t-test, two-tailed) as indicated.

One of the unique characteristics of cancer cells is that they do not need to be anchored to a surface and have the ability to grow in soft agar. To evaluate whether AZD3463 could inhibit the colony-forming ability of NB cell lines, soft agar assays were performed with a panel of six NB cell lines. In this assay, the result showed that AZD3463 inhibited anchorage-independent colony formation of all tested NB cells, including both ALK WT and mutant, in a dose-dependent manner (Fig. 2A,B). Our data indicates that AZD3463 effectively blocks both anchorage-dependent and independent colony formation of NB cells.

### AZD3463 effectively inhibits ALK-mediated PI3K/AKT/mTOR signaling and induces apoptosis and autophagy in NB cells

**Figure 3:**
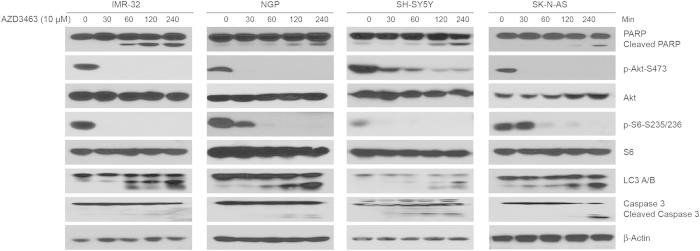
AZD3463 inhibits the downstream signaling pathway of ALK, PI3K/AKT/mTOR, and induces apoptosis and autophagy in NB cells. IMR-32, NGP, SH-SY5Y and SK-N-AS cells were treated with 10 μM of AZD3463 for various time points (0–4 hrs), subjected to SDS-PAGE, and then immunoblotted with PARP, p-Akt, Akt, p-S6, S6, Caspase 3, LC3A/B, and β-Actin antibodies.

PI3K/AKT/mTOR and JAK/STAT are the most important signaling pathways downstream of ALK and play an important role in cell growth in many tumor types. Previous studies have revealed that while involvement of the STAT pathway is rare in NB, ALK gain-of-function mutations signal mainly through PI3K/AKT/mTOR pathways^24^. If we block the activation of ALK, this should lead to inhibition of the PI3K/AKT/mTOR pathway. To investigate whether this inhibitory effect could be induced by AZD3463 in human NB cell lines, four NB cell lines were treated with AZD3463 for various lengths of time *in vitro*. Western blots were followed to analyze the pathway activity and the protein expression level of its downstream effectors. As expected, in four tested NB cell lines, the phosphorylation of Akt Ser473 and RPS6 Thr235/236, were potently inhibited or totally abolished by AZD4363 (Fig. 3). The results showed that AZD3463 significantly inhibited PI3K/AKT/mTOR signaling within 30 minutes, as indicated by the decrease in the phosphorylation level of the downstream effectors.

We also noticed that the AZD4363 induced PARP and caspase 3 cleavages in both ALK WT and mutant cell lines within 2–4 hours. Moreover, it has been reported that mTOR is the main negative regulator of autophagy^30^. Indeed, AZD3463 induced cleavage of the autophagy marker LC3 A/BΙΙ within four hours of treatment. These results indicate that AZD3463 inhibited NB cell proliferation through the induction of both apoptosis and autophagy.

### ALK inhibitor AZD3463 significantly enhances the cytotoxic effects of Dox on NB cells

**Figure 4:**
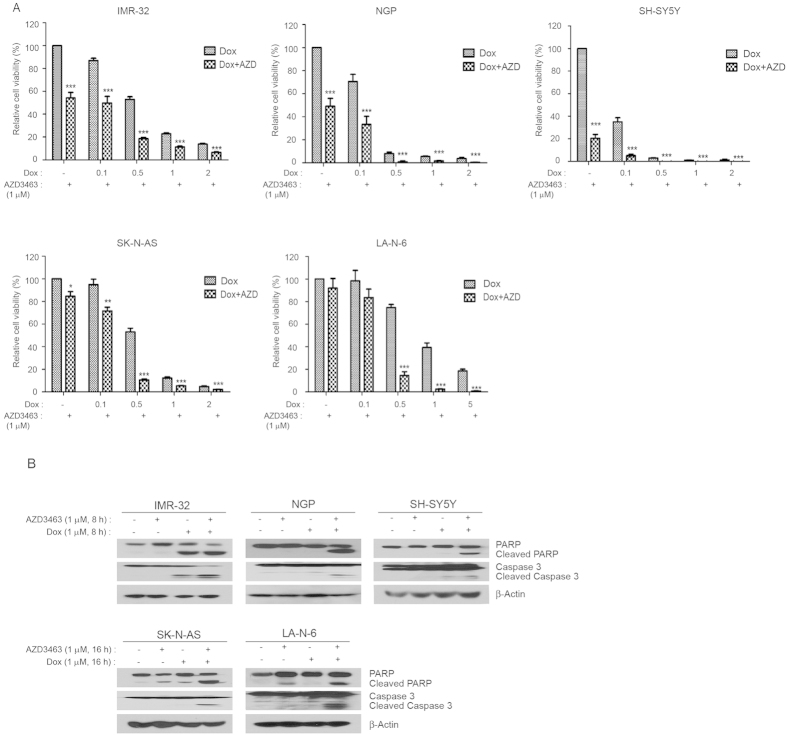
AZD3463 enhances the cytotoxic effect of Dox on NB cell lines. (**A**) IMR-32, NGP, SH-SY5Y, SK-N-AS, and LA-N-6 cells were seeded in 96-well plates and were incubated with the indicated concentrations of Dox plus DMSO or 1 μM of AZD3463 for 48 hrs. Cell viability was then measured by CCK-8 assay. (**B**) IMR-32, NGP, SH-SY5Y, SK-N-AS, and LA-N-6 cells were treated with either Dox (1 μM) alone, AZD3463 (1 μM) alone, or a combination for varying durations (0–8 hrs or 0–16 hrs), subjected to SDS-PAGE, and immunoblotted with PARP and Caspase-3 antibodies. β-Actin antibodies were used as a loading control for whole cell extracts in all samples.

Monotherapies are rarely effective in the treatment of high-risk NB due to chemoresistance. Thus, we evaluated the combination effect of AZD3463 and Dox by using a panel of five NB cell lines, including ALK WT, ALK mutant cell lines, and the chemoresistant LA-N-6 cell line. We found that the co-culture with AZD3463 enhanced the cytotoxicity of Dox on all five cell lines tested (Fig. 4A). Consistently, ALK inhibition also significantly increased Dox-induced PARP cleavage and caspase 3 cleavage, indicating increased cell death in the treated cells (Fig. 4B). More importantly, besides the potent enhanced cytotoxic effect of AZD3463 with Dox on NB cells, AZD3463 was even more effective than Dox when comparing induced cleavage of PARP and caspase 3 levels in SK-N-AS and LA-N-6 cells (Fig. 4A,B).

### AZD3463 shows anti-tumor efficacy in both ALK WT and F1174L mutant orthotropic xenograft mouse models of NB

**Figure 5:**
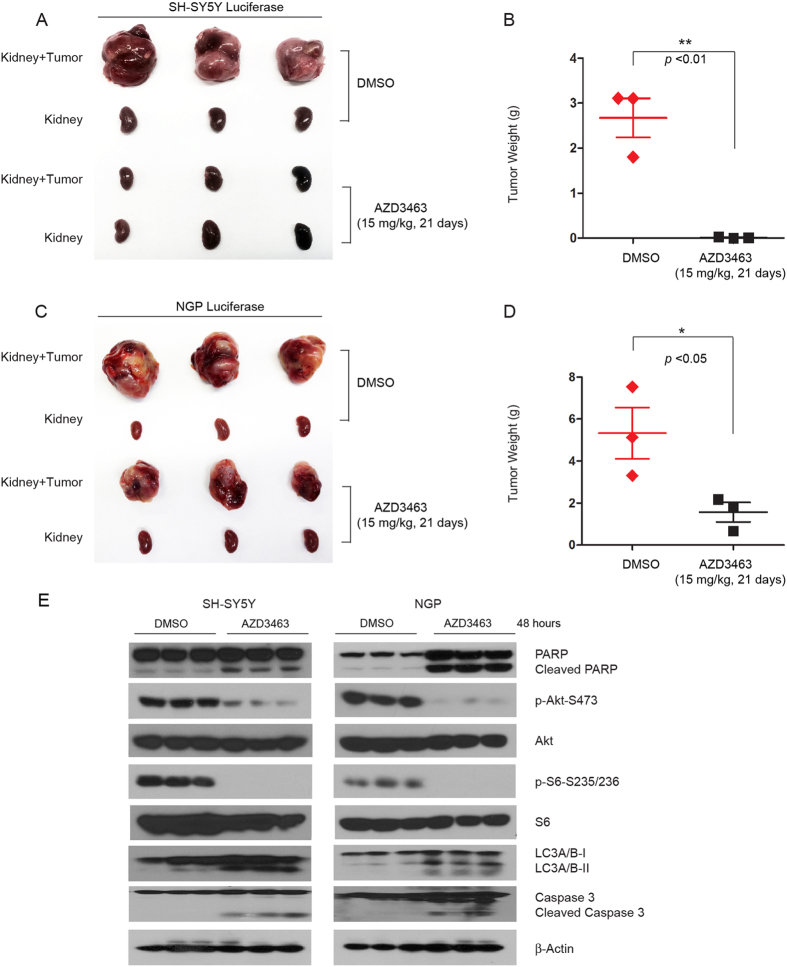
AZD3463 inhibits tumor growth in different orthotopic NB xenograft mouse models. (**A**) Photos of SH-SY5Y xenografted tumors and the corresponding kidney controls from DMSO control group and AZD3463 treatment group (15 mg/kg) were taken at the end of treatment (21 days). (**B**) SH-SY5Y derived tumor weights from control (N = 3) and treatment groups (N = 3) were presented as the mean with SDs. *P* < 0.01 (**) (Student’s t-test, two-tailed) was indicated. (**C**) Photos of NGP xenografted tumors and the corresponding kidney controls from DMSO control group and AZD3463 treatment group (15 mg/kg) were taken at the end of treatment (21 days). (**D**) NGP-derived tumor weights from control (N = 3) and treatment groups (N = 3) were presented as the mean with SDs. *P* < 0.05 (*) (Student’s t-test, two-tailed) was indicated. (**E**) The mice bearing SH-SY5Y and NGP xenograft tumors for 4 weeks were treated with DMSO or 15mg/kg of AZD3463 by intraperitoneal injection twice in 48 hrs, and then the tumors were harvested and subjected to SDS-PAGE and immunoblotted with indicated antibodies. β-Actin was detected as a loading control.

Based on the cytotoxic effects of AZD3463 on NB cells *in vitro*, we proceeded to assess the drug’s effect on inhibiting tumor growth in orthotopic xenograft mouse models of NB. In this set of *in vivo* experiments, NGP and SH-SY5Y cells with stable luciferase gene expression were separately implanted into the left kidneys of the nude mice. At the end of the AZD3463 treatment, the xenograft tumors from control and treatment groups were dissected and weighed. Significant tumor growth inhibition was observed in both AZD3463 treatment groups (SH-SY5Y and NGP) compared with the control groups. Treatment in SH-SY5Y xenograft mice with AZD3463 resulted in almost complete tumor regression and significant regression was observed in NGP xenograft mice (Fig. 5A,C). After the mice had been bearing the SH-SY5Y and NGP tumors for 4 weeks, the mice were treated with either AZD3463 or DMSO via intraperitoneal injection for 48 hours. Then we examined the effect of AZD3463 on the PI3K/AKT/mTOR signaling in the tumor tissues and found that AZD3463 efficiently blocked Akt and RPS6 phosphorylation and induced the cleavage of PARP, caspase 3, and LC3 A/BΙΙ (Fig. 5E) *in vivo*. These results suggest that AZD3463 can effectively induce apoptosis and autophagy as a single agent in orthotopic xenograft mouse models of NB.

## Discussion

The rapid FDA approval of the first-generation ALK inhibitor crizotinib for first-line treatment of ALK-positive NSCLC in 2011 was a milestone in the history of clinical development of small molecular inhibitors to treat cancer. Activating mutations in ALK remain the most common targetable mutations identified in NB to date, opening new opportunities for novel therapeutic strategies against this often-fatal childhood cancer of the sympathetic nervous system^31^. In the past few years, the development of new drugs which block the ALK pathway by a mechanism different from that of crizotinib could represent a useful approach to circumvent the problems associated with crizotinib. To overcome this issue, we investigated the effect of AZD3463, a novel ALK inhibitor, on NB growth. We found that AZD3463 could effectively block both WT and mutant ALK signaling *in vitro* through blocking the downstream PI3K/AKT/mTOR pathway in all four tested NB cell lines in as short as a few minutes. This suggests that this inhibitor has a high cytotoxic activity, accompanied with decreased cell proliferation, attenuated colony forming ability, and increased apoptosis. Notably, one of the tested cell lines, SH-SY5Y, harbors the F1174L mutation, clinically one of the most common mutations that exhibits resistant to both crizotinib and ceritinib. These data suggest that AZD3463 can efficiently inhibit the activity of both WT and mutant ALK. More importantly, AZD3463 significantly inhibited the growth of NB tumors with WT and mutant ALK in orthotopic mouse models. Our results suggest that the anti-tumor effect in the SH-SY5Y NB orthotopic xenograft mouse model is much more effective than that in the NGP mouse model. Treatment of SH-SY5Y xenograft mice with AZD3463 for 21 days resulted in complete tumor regression while a significant regression was observed in NGP xenograft mice. One possible explanation is that NB cells with ALK aberration (mutation or amplification) are ALK addictive^20^,^32^. Moreover, NGP is well known to be a more malignant NB cell characterized with MYCN amplification but without ALK amplification. Considering that its cytostatic activity leads to tumor growth retardation, AZD3463 shows great clinical potential and may play an important role in the treatment of NB patients in the future.

We also noticed that mutations or amplifications of ALK were found only in a fraction of NB cases. What about the remaining others? It remains a fascinating question for future studies whether non-mutated or non-amplified ALK contributes significantly to NB tumorigenesis and what is the mechanism of this contribution. Interestingly, knockdown of ALK gene in NB cells bearing the WT copy leads to apoptosis and cell death^11^,^14^, implying a wider role for the kinase in this disease. Our inhibition of ALK in four WT NB cell lines confirmed this observation. It suggests that ALK inhibitors, such as AZD3463, can be effective not only in the 15% of patients with ALK activating mutation or abnormal amplification but also in patients with WT tumors.

A significant increase of autophagy was also observed *in vitro* and *in vivo* when NB cells were treated with AZD3463. It is very interesting that autophagy was literally characterized as both cytoprotective and cytotoxic factors in cancer cells. Although it is still too early to make a final conclusion for the role of autophagy in cell death and cancer, nearly half of the studies have shown that autophagy is essential for the efficient killing of tumor cells when treated with anticancer therapies^33^, and many natural compounds and extracts with anti-cancer activity could initiate and regulate autophagy in tumor cells^34^. Accumulating data indicates that apoptosis and autophagy can be observed at the same time in tumors including NB and have a close relationship with PI3K/AKT/mTOR pathway. However, how apoptosis and autophagy interactions remain largely unclear^35^,^36^,^37^,^38^,^39^,^40^,^41^,^42^,^43^. Here our study reveal that AZD3463 induces autophagy, as well as apoptosis, in NB cells and xenograft mouse model tissues at an early time. It is likely that autophagy may have a synergistic effect with apoptosis. Since mTOR has been reported as the major negative regulator of autophagy in tumors and AZD3463 could effectively block the activity of ALK and its downstream mTOR pathway, it is not surprising that autophagy was observed when the NB cells and tumors in mice were treated with AZD3463. There is no doubt that there are multiple types of crosswalks between the apoptosis and autophagy activities in tumors.

Another critical issue frequently raised in the clinical setting is the drug resistance and toxicity of single or combination chemotherapy. Novel therapeutic options are urgently needed to overcome chemoresistance and improve patient survival. Activating mutation in ALK is the most frequent mutation identified in NB to date. Because long time use of even the most effective inhibitor alone would ultimately generate resistance, a blend of inhibitors could sensitize tumors to various forms of therapy. As shown, though NB cells showed higher sensitivity to AZD3463 in our assays, NB cells were further sensitized by a combination of AZD3463 and Dox treatment. Interestingly, we observed that AZD3463 is more effective in inhibiting cell proliferation of a chemo-resistant NB cell line LA-N-6 than doxorubicin. Furthermore, AZD3463 enhanced the cytotoxic effect of doxorubicin in LA-N-6 cells. It is possible that combination therapy using AZD3463 may sensitize NB tumor cells to chemotherapy and thus decrease drug toxicity. A study showed combined treatment of crizotinib and an mTOR inhibitor led to reduced tumor growth and prolonged survival in ALK F1174L/MYCN-positive models compared to single agent treatment^44^. Since AZD3463 alone can inhibit ALK signaling, we can combine it with other receptor tyrosine kinase inhibitors to increase efficacy for NB patients. Another paper reported that in lung cancer, EGFR, Her2 and P2Y genes are enriched in crizotinib-resistant tumors^3^. Thus in future studies, besides the use of ALK inhibitors; we will develop strategies that inhibit multiple targets to model better treatment options for NB patients. In summary, by using a panel of NB cell lines and *in vivo* orthotopic mouse models of NB, we provide compelling evidence that AZD3463 is able to induce tumor regression as a single agent and in combination with other chemotherapeutic agents. This study suggests that AZD3463 might serve as an effective drug in the design of potential clinical trials for patients with recurrent or refractory NB, especially ones who are resistant to crizotinib.

## Materials and Methods

### Antibodies and Reagents

ALK inhibitor AZD3463 was purchased from Apexbio (A8620, ChemieTek, Indianapolis, IN, USA). Doxorubicin (Dox, D1515) and anti-β-Actin antibodies (A2228) were purchased from Sigma (Sigma-Aldrich Corp, St. Louis, MO, USA). The remaining antibodies—rabbit monoclonal pSer473 Akt (4060S), rabbit monoclonal Akt (9272), rabbit monoclonal pSer235/236 S6 ribosomal protein (4858S), rabbit monoclonal S6 ribosomal protein (2217S), rabbit monoclonal PARP (9532S), rabbit polyclonal caspase 3 (9662S), rabbit polyclonal LC3 A/B (4108S), and anti-Mouse (7076S) or anti-Rabbit (7074S) IgG—were purchased from Cell Signaling Technology (Danvers, MA, USA).

### Cell Lines and Cell Culture

Four ALK wild type human NB cell lines—SK-N-AS, IMR-32, NGP, NB-19—and one ALK mutant human NB cell line—SH-SY5Y were cultured in RPMI Medium 1640 (Lonza, Walkersville, MD, USA) supplemented with 10% (v/v) heat-inactivated Fetal Bovine Serum (FBS) (SAFC Biosciences, Lenexa, KS, USA), 100 units/mL penicillin, and 100 μg/mL streptomycin. The chemoresistant NB cell line LA-N-6 was grown in RPMI containing 20% (v/v) heat-inactivated FBS, 100 units/mL penicillin, and 100 μg/mL streptomycin. All cells were maintained at 37 °C in a humidified incubator with 5% CO_2_. All experiments were performed with cells under exponential growth conditions. The SH-SY5Y cell line with a stable expression of luciferase was generated by transfection with a pcDNA3 luciferase expression plasmid into the cells. A stable cell line was established after 10 days of applying 800 μg/ml G418 selection (Enzo Life Sciences, Farmingdale, NY, USA).

### Cell Viability Assay

Cell viability assays were performed using the Cell Counting Kit-8 (CCK-8, WST-8[2-(2-methoxy-4-nitrophenyl)-3-(4-nitrophenyl)-5-(2, 4-disulfophenyl)-2 H-tetrazolium, monosodium salt]) (Dojindo Laboratories, Rockville, MA, USA). Cells were plated and grown in 96-well clear-bottom plates starting at 1 × 10^4 ^cells/well. After 24 hrs of incubation, media was changed and increasing concentrations of AZD3463, Dox, or their combinations were added to the wells and the cells were then incubated at 37 °C for 48 or 72 hrs. Then a mixture of 10 μL of CCK-8 and 190 μL of RPMI with 10% FBS was added into each well. After 1 hour of incubation, the absorbance was measured at 450 nm using a microplate reader. Each experiment was performed in replicates of six and background reading of the media was then subtracted from each well to standardize the results.

### Cell Imaging

A total of six NB cell lines were separately seeded in 96-well plates at appropriate concentrations. After 48 hrs or 72 hrs of treatments with indicated concentrations of AZD3463, Dox, or their combinations, cell morphologies were observed and captured using an optical microscope. Each result was performed in triplicate.

### Colony Formation Assay

The soft agar assay was performed as described previously^4^,^45^. Briefly, a 5% (w/v) base agar layer was made by adding agar (214220, Difco Laboratories, Detroit, MI, USA) into distilled water, and then the mixture was autoclaved for 50 min and cooled in a 56 °C water bath. This solution was then mixed with RPMI with 10% FBS to a final concentration of 0.5%. To apply the bottom agar layer, 2 mL of the 0.5% agar/RPMI solution were added to each well then cooled until semi-solid. For the top agar layer, each NB cell line was counted and added to 1.5 ml 0.3% agar at 1 × 10^4 ^cells/well along with the indicated concentrations of AZD3463. Cells were grown at 37 °C for 2 to 3 weeks, then stained with 500 μL of 0.005% crystal violet (C3886, Sigma). Images were captured by microscope and colonies were counted after 4 hours. Each assay was performed in triplicate.

### Immunoblotting

After each drug treatment, cells were washed twice with ice cold PBS and then lysed on a rotator in 4 °C for 30 min in a cooled RIPA buffer (50 mM Tris-HCl at pH 7.4, 150 mM NaCl, 1 mM EDTA, 1% NP-40, 0.25% sodium deoxycholate, 1 mM phenylmethylsulfonyl fluoride, 1 mM benzamidine, 10 μg/mL leupeptin, 1 mM dithiothreitol, 50 mM sodium fluoride, 0.1 mM sodium orthovanadate, and phosphatase inhibitor cocktail 2 and 3 (p5726 and p0044, Sigma)). After centrifuging at 13,000 rpm for 15 min, supernatants were used as cell lysates. Protein concentrations were measured using Bradford reagent (Bio-Rad Laboratories, Hercules, CA, USA). Each sample was mixed 3:1 (v/v) with 4× loading buffer and heated to 100 °C for 6 min. Lysates were then separated by SDS-PAGE, transferred to polyvinylidence fluoride (PVDF) membranes (Bio Rad), blocked with 5% milk or BSA for one hour at RT (25 °C), and probed with appropriate dilutions of indicated primary antibodies overnight at 4 °C. The membranes were incubated with anti-mouse or anti-rabbit IgG conjugated with horseradish peroxidase at room temperature for 1 hr. ECL-Plus Western detection system (GE Health Care, Buckinghamshire, UK) was then used for chemiluminescent visualization. β-Actin was used as a loading control for whole cell extracts.

### Flow Cytometry and Propidium Iodide (PI) Staining Assay

The experiment was performed as described previously^46^. Briefly, NB cell lines were seeded in 6 cm dishes and treated with 10 µM of AZD3463 for 2–4 hours. Cells were trypsinized, resuspended in RPMI 1640 medium, and centrifuged at 500 g for 5 min at 4 °C. Cells were then washed with cold 1× PBS twice, and resuspended at a density of 1 × 10^6 ^cells/ml in 1× binding buffer (51-66121E; BD Biosciences, San Jose, CA, USA). Then 100 μl of non-fixed cell suspension was transferred into a new tube and 5 μl of 50 μg/mL PI staining solution (51-66211E; BD Biosciences) was added into the tube. The tubes were gently vortexed and incubated for 15 min at RT (25 °C) in the dark. After adding the additional 400 μl of 1× binding buffer, the samples were analyzed by flow cytometry within 1 hr. As viable cells with intact membranes resist PI staining, only the membranes of dead cells are subject to PI staining. Unstained cells were used as a negative control and untreated cells were used as a control for treated cells. Then analysis was performed on a LSR-II flow cytometer (BD Biosciences) using BD FACDiva software v. 6.0.

### Orthotopic Mouse Model of NB

Five to six-week-old female athymic Ncr nude mice were purchased from Taconic (Taconic, Hudson, NY, USA) and maintained under barrier conditions (pathogen-free conditions provided by plastic cages with sealed air filters). The preclinical mouse model of NB was established using orthotopic (intrarenal) implantation of the NB cells. Briefly, a transverse incision was created over the left flank of the nude mouse and 1.5 × 10^6^ human luciferase-transduced SH-SY5Y or NGP cells suspended in 0.1 ml of PBS were surgically injected into the left renal capsule and towards the superior pole of the left kidney of the nude mice. After allowing the cells to engraft for 2 to 3 weeks, mice bearing tumors with similar sizes (using bioluminescent imaging to monitor tumor growth) were randomly divided into two groups: a DMSO control group and an AZD3463 treatment group. Both control and experimental groups contained six mice each. Three mice from each group were selected and treated with DMSO or AZD3463 over a 48 hour period via15 mg/kg intraperitoneal (i.p.) injection once daily for 2 days. Tumors were then harvested for WB analysis. The rest were treated for 21 days (15 mg/kg by intraperitoneal (i.p.) injection once daily). At the end of the treatment, all mice were sacrificed. Tumors and the right kidneys (control) were harvested and weighed. All mice were handled according to protocols approved by Institutional Animal Care and Use Committee of Baylor College of Medicine.

### Statistical Analysis

All values were presented as mean ± standard deviation (SD). A two-tailed Student’s t-test was used to determine the statistical significance of *in vitro* and *in vivo* assay between the control and drug treatment groups. Each assay was repeated at least twice and representative results were presented. *P* < 0.05 was considered to be statistically significant.

## Additional Information

**How to cite this article**: Wang, Y. *et al.* Novel ALK inhibitor AZD3463 inhibits neuroblastoma growth by overcoming crizotinib resistance and inducing apoptosis. *Sci. Rep.* **6**, 19423; doi: 10.1038/srep19423 (2016).
